# A Potent Autophagy Inhibitor (Lys05) Enhances the Impact of Ionizing Radiation on Human Lung Cancer Cells H1299

**DOI:** 10.3390/ijms20235881

**Published:** 2019-11-23

**Authors:** Lucie Cechakova, Martin Ondrej, Vojtech Pavlik, Petr Jost, Dana Cizkova, Ales Bezrouk, Jaroslav Pejchal, Ravi K. Amaravadi, Jeffrey D. Winkler, Ales Tichy

**Affiliations:** 1 Department of Radiobiology, Faculty of Military Health Sciences, University of Defense in Brno, 500 01 Hradec Kralove, Czech Republic; lucie.cechakova@unob.cz (L.C.); martin.ondrej@unob.cz (M.O.); 2 Cell Physiology Research Group, Contipro, 561 02 Dolni Dobrouc, Czech Republic; vojtech.pavlik@contipro.com; 3 Institute of Dermatology, Third Faculty of Medicine, Charles University in Prague, 100 00 Prague 10-Vinohrady, Czech Republic; 4 Department of Toxicology and Military Pharmacy, Faculty of Military Health Sciences, University of Defense in Brno, 500 01 Hradec Kralove, Czech Republic; petr.jost@unob.cz (P.J.); jaroslav.pejchal@unob.cz (J.P.); 5 Department of Histology and Embryology, Faculty of Medicine in Hradec Kralove, Charles University in Prague, 500 01 Hradec Kralove, Czech Republic; CizkovaD@lfhk.cuni.cz; 6 Department of Medical Biophysics, Faculty of Medicine in Hradec Kralove, Charles University in Prague, 500 01 Hradec Kralove, Czech Republic; BezroukA@lfhk.cuni.cz; 7 Abramson Cancer Center and Perelman School of Medicine, University of Pennsylvania, Philadelphia, PA 19104, USA; Ravi.Amaravadi@uphs.upenn.edu (R.K.A.); winkler@sas.upenn.edu (J.D.W.)

**Keywords:** autophagy, ionizing radiation, Lys05, cancer, non-small cell lung carcinoma

## Abstract

Autophagy inhibition through small-molecule inhibitors is one of the approaches to increase the efficiency of radiotherapy in oncological patients. A new inhibitor—Lys05—with the potential to accumulate within lysosomes and to block autophagy was discovered a few years ago. Several studies have addressed its chemosensitizing effects but nothing is known about its impact in the context of ionizing radiation (IR). To describe its role in radiosensitization, we employed radioresistant human non-small cell lung carcinoma cells (H1299, p53-negative). Combined treatment of H1299 cells by Lys05 together with IR decreased cell survival in the clonogenic assay and real-time monitoring of cell growth more than either Lys05 or IR alone. Immunodetection of LC3 and p62/SQSTM1 indicated that autophagy was inhibited, which correlated with increased *SQSTM1* and decreased *BNIP3* gene expression determined by qRT-PCR. Fluorescence microscopy and flow cytometry uncovered an accumulation of lysosomes. Similarly, transmission electron microscopy demonstrated the accumulation of autophagosomes confirming the ability of Lys05 to potentiate autophagy inhibition in H1299 cells. We report here for the first time that Lys05 could be utilized in combination with IR as a promising future strategy in the eradication of lung cancer cells.

## 1. Introduction

Lung cancer and non-small cell lung carcinoma (NSCLC) in particular represents the leading cause of cancer-related mortality worldwide [1,2]. Because of conditions making progressed tumor inoperable, the majority of patients are treated by radiation therapy (RT). However, the radioresistance of NSCLC contributes to unsatisfactory recurrence, driving an escalating interest in the improvement of the treatment [1,2].

One of the options, which could increase the efficacy of RT, is the modulation of autophagy. Autophagy is an intracellular mechanism enabling the eradication of damaged organelles and proteins in order to protect cells from stress triggered by various stimuli, including IR. It utilizes lysosomes as the tools for the degradation of substrates [3]. Typically, the double-membrane autophagosome (phagophore) is formed in order to absorb molecules to be degraded. This process consists of induction, nucleation, elongation, and maturation. While the main regulator of nucleation is phosphoinositide 3-kinase (PI3K), autophagosome formation relies on ubiquitin-like conjugation systems (ATG12 and LC3). Finally, lysosome fuses with autophagosome to form autophagolysosome and triggers the release of the acidic lysosomal hydrolases, which degrade the cytosolic substrates [4]. Notwithstanding, autophagy is a somewhat controversial process. Current literature describes autophagy as a mechanism with two opposing roles in tumor cells. In the early stages of cancer development, autophagy contributes to the suppression of tumor growth. On the other hand, in the advanced stages of cancer development, it acts as a promoter of carcinogenesis [4]. Since autophagy has been believed to be a protective mechanism in developed cancers, inhibition of this process seems to be a promising strategy to enhance the efficacy of RT.

Lys05—a new autophagy inhibitor—was reported for the first time by McAfee et al. several years ago. It is a dimeric form of a well-established autophagy inhibitor chloroquine (CQ). Mechanistically, Lys05 similarly as CQ and hydroxychloroquine (HCQ) has the potential to accumulate within lysosomes and to block autophagy even more effectively than CQ or HCQ. Moreover, it exhibits the most potent anti-tumor activity as a single agent among the three above-mentioned inhibitors both in vitro and in vivo [5]. Although Lys05 is a promising agent, no study examined its sensitizing effect in combination with IR so far.

The aim of our study was to sensitize the radioresistant H1299 cells toward IR using the specific autophagy inhibitor Lys05 and to describe the underlying molecular mechanisms involved in this process. For comparison of the Lys05-induced anti-proliferative effect, we used two established autophagy inhibitors: Bafilomycin A1 (Baf) and Spautin-1. Baf is an inhibitor with a similar mechanism of action as Lys05. It inhibits the final step of the autophagic process—a fusion between autophagosomes and lysosomes [6]. Spautin-1 works differently. It specifically targets the Beclin-1 subunit of the PI3K complex and causes its degradation [7,8]. It has been shown to enhance IR-induced cell death [9] and to reduce tumor growth in mice xenografts without side effects [8]. CQ or HCQ has not been used as controls in our study since head-to-head comparison with Lys05 had previously been described by McAfee et al. [5].

The combination of IR and Lys05 led to more efficient eradication of radioresistant cells compared to either agent alone. These results suggest that Lys05 might provide an efficient tool for the radiosensitization of cancer cells and could be utilized as a promising future strategy in radiotherapy of lung cancer.

## 2. Results

### 2.1. Treatment of H1299 Cells by Autophagy Inhibitors and IR Causes a Decrease in Proliferation in a Concentration- and Dose-Dependent Manner

The xCELLigence system enables non-invasive real-time monitoring of cell characteristics such as viability, proliferation, and adhesion. This assay is performed in microtiter plates with gold electrodes on the bottom of each well and changes in electrical impedance are measured. Cell culture was monitored every 30 min for five days. Obtained data were expressed as cell index. The higher the cell index value, the more cells are attached to the electrodes [10,11].

Initially, we performed experiments with different concentrations of inhibitors and doses of IR. Spautin-1 was selected as a control in proliferation evaluating the methods. In order to compare the overall inhibition effect of the studied autophagy inhibitor—Lys05, we used the inhibitor possessing a different mechanism of action (unlike for further experiments with autophagosome accumulation). For the sake of simplicity, we compared our results with only one inhibitor in each method. We observed a concentration-dependent decrease in cell proliferation in the groups treated by both Spautin-1 and Lys05 alone. Similarly, the higher the dose of IR, the more pronounced decrease in proliferation was achieved (data are shown in Figure S1). Based on these results, we selected 2 µM Lys05 or 2 µM Spautin-1 as the lowest concentration and the lowest dose of 2 Gy (actually a D0 that causes cell death in 63% of the cell population) for the further experiments. The higher concentration and doses would probably lead to more intensive effects, but our effort was driven by the radiosensitization hypothesis—the combined effect of the lowest doses of agents possibly leading to a mutual potentiation of the anti-proliferative effect.

**Figure 1:**
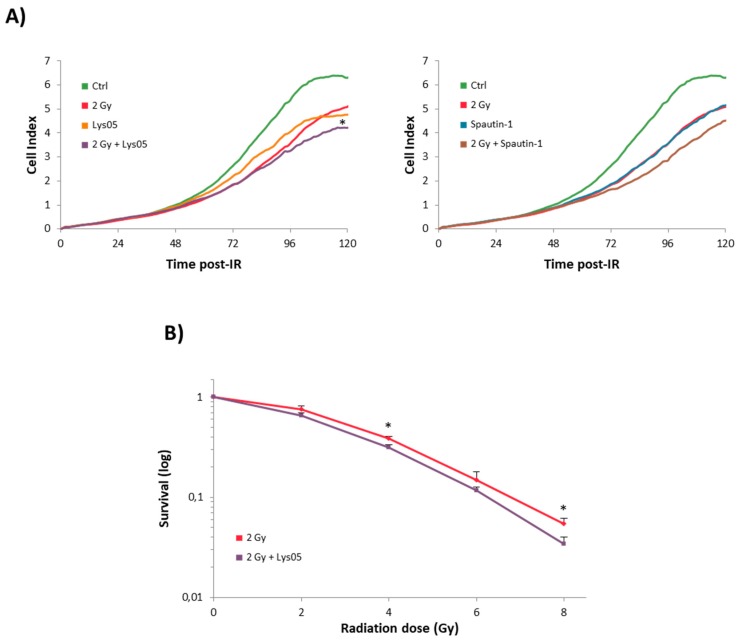
Autophagy inhibitors caused a decline in H1299 cell proliferation. The H1299 cells were treated either by ionizing radiation (IR) or inhibitor alone or by their combination. Lys05 or Spautin-1 in 2 µM concentration was added to the cells one hour prior to irradiation (2, 4, 6, and 8 Gy). (**A**) Cell proliferation was measured by the xCELLigence system and recorded every 30 min for six days. * Significant difference compared to control (*p*-value ≤ 0.05) as a result of one experiment performed in triplicate. (**B**) Clonogenic assay displays average values ± SD from three independent experiments in triplicates counted by eye after the 10-day incubation period. * Significant difference compared to solely irradiated group (*p*-value ≤ 0.05).

The combination of 2 Gy and 2 µM Lys05 or Spautin-1 resulted in a more pronounced decrease in proliferation than single treatment (Figure 1A).

### 2.2. Lys05 Renders H1299 Cells More Sensitive to IR

The real-time monitoring provided valuable insight into cell-culture dynamics after the treatment. However, taking into account its limitations, we assessed the influence of Lys05 on H1299 cells in terms of radiosensitization by a more traditional radiobiological approach. Hence, the clonogenic assay was used.

H1299 cells were treated by Lys05 (2 µM) one hour prior to irradiation (2, 4, and 8 Gy). The survival fraction decreased in a dose-dependent manner. Administration of Lys05 resulted in statistically significant suppression of clonogenic ability compared to the cells exposed to IR only (Figure 1B). These results support our conclusion that the inhibition of autophagy by Lys05 enhanced the cytotoxicity of IR. Complete data are shown in Supplementary Figure S1.

### 2.3. The Combination of Inhibitors with Irradiation Shows an Additive Effect

Although the combined treatment of H1299 cells resulted in the most pronounced decrease in proliferation, we analyzed the combination index (CI) in order to determine the estimation of the extent of synergy or antagonism between each inhibitor and IR. CI was calculated based on the Chou–Talalay method (briefly reviewed in [12]) using the CompuSyn software. According to the CI value description published by Hernández et al. CI values under the 0.9 indicate synergism, CI values between 0.9 and 1.2 indicate additive effect, and CI values higher than 1.2 indicate antagonism [13].

**Figure 2:**
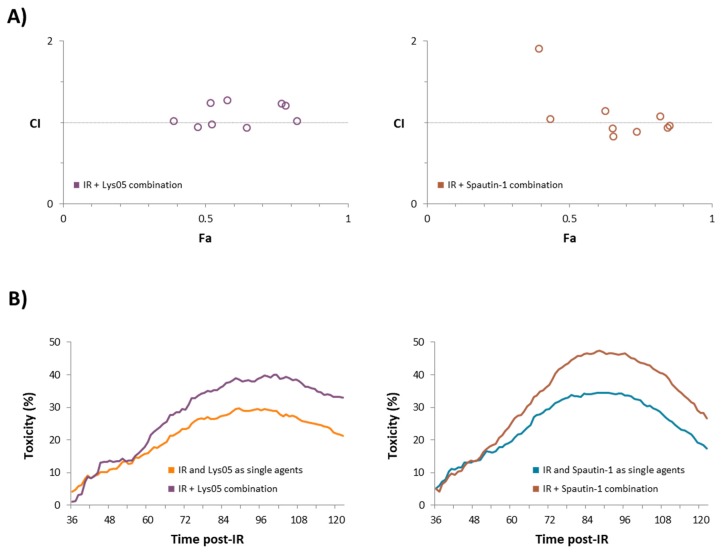
The combination of inhibitors with irradiation shows an additive effect. Data for these computations were derived from real-time monitoring of cell characteristics measured by the xCELLigence system. (**A**) Combination index was calculated based on the Chou–Talalay method using the CompuSyn software. The relevant Fa-CI plots are shown according to the data available in Table 1. Fa = fraction affected/toxicity and CI = combination index. (**B**) The time-dependent combined toxicity level was calculated based on the real-time monitoring data with a selected concentration of 2 µM for each inhibitor and a radiation dose of 2 Gy. The time-dependent combined toxicity level shows the ratio of the sum of anti-proliferative effects of each inhibitor and IR as single agents relative to the control compared with the ratio of the combination of inhibitor and IR relative to the control. The combined toxicity values were plotted versus time in hours.

We calculated the CI based on the anti-proliferative effects of the inhibitors and IR observed at a time point when the changes in proliferation were most prominent—100 hours after irradiation. Our data indicate the additive effects of both inhibitors and IR (Table 1 and Figure 2A).

Considering that CI evaluates the extent of synergism/antagonism only at one point in time, we compared the ratio of the sum of anti-proliferative effects of each inhibitor and IR as single agents relative to the control with the ratio of the combination of inhibitor and IR relative to the control. The concentration of each inhibitor and the dose of irradiation selected for this measurement were 2 µM and 2 Gy, respectively.

Based on this calculation, we observed stronger anti-proliferative effects provided by the combination of each inhibitor with IR depending on the time than their sum when acting as single agents (Figure 2B).

### 2.4. Lys05 Does Not Affect the Apical Parts of mTOR Signaling but Results in Increased LC3-II and p62/SQSTM1

To evaluate how Lys05 interferes with autophagy signaling pathways, we used two methods: (i) Western blotting (WB) and (ii) real-time quantitative reverse transcription PCR (qRT-PCR).

**Figure 3:**
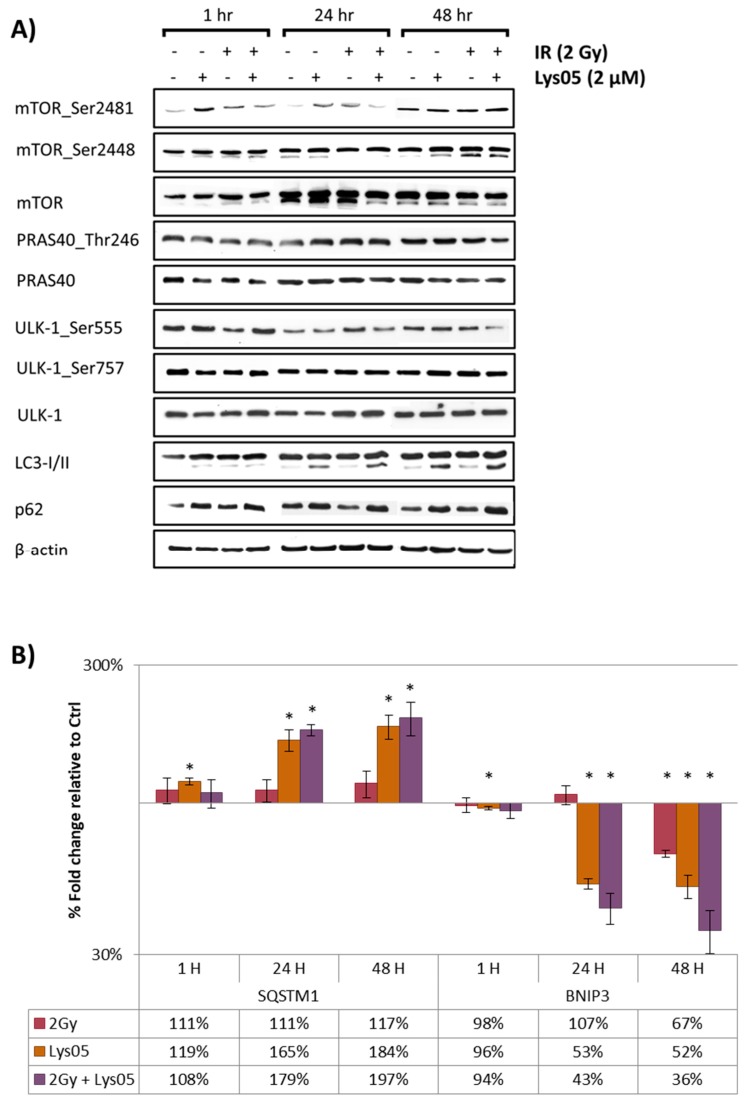
Administration of Lys05 blocks autophagosome-lysosome fusion. The H1299 cells were treated either by IR or the inhibitor alone or by their combination. Lys05 in 2 µM concentration was added to the cells one hour prior to irradiation (2 Gy). Data were analyzed one, 24, and 48 h after irradiation. (**A**) Western blot analysis of phosphorylated and non-phosphorylated proteins following treatment by IR and Lys05. Full-length blots are presented in Supplementary Figure S2. (**B**) qRT-PCR analysis of the expression of genes *SQSTM1* and *BNIP3*. Data represent the mean of the percentage fold change (±SD) relative to control based on three independent experiments performed in duplicate. * Significant difference compared to control (*p*-value ≤ 0.05).

Using WB, we examined the level of mammalian target of rapamycin (mTOR) and its phosphorylated forms (serine 2448 and 2481), Unc-51 like autophagy activating kinase 1 (ULK-1) and its phosphorylated forms (serine 555 and 757), and proline-rich Akt substrate of 40 kDa (PRAS40) and its phosphorylated form (threonine 246) one, 24, and 48 h after irradiation. We did not notice any significant changes in any of the groups or time-points indicating that Lys05 does not affect the initial stage of autophagy signaling (Figure 3A).

Further on, we examined the microtubule-associated protein 1A/1B-light chain 3 (LC3) and ubiquitin-binding protein p62 (p62/SQSTM1). Administration of Lys05 and/or irradiation (2 Gy) led to an increase in LC3-II protein form and also to a non-significant increase in protein p62/SQSTM1 in the groups of cells treated either by Lys05 alone or by its combination with IR, especially after 48 h (Figure 3A). Increased LC3II/I ratio together with elevated levels of p62/SQSTM1 demonstrated the inhibition of autophagy via blockade of autophagosome-lysosome fusion [14].

As Western blotting is considered only a semi-quantitative approach, we utilized the microarray assay to perform a complex screening of the effects of Lys05 on the level of mRNA (data are available in Supplementary Tables S1 and S2, and GEO repository: GSE138650), which led us to further examination of two particular genes using qRT-PCR: *SQSTM1* and Bcl2 interacting protein 3 (*BNIP3*). These genes are well-known to be associated with autophagy [15,16]. Administration of Lys05 and/or irradiation (2 Gy) led to a significant increase in *SQSTM1* expression and decrease in *BNIP3* expression in the groups of cells treated either by Lys05 alone or by its combination with IR both after 24 and 48 h (Figure 3B).

### 2.5. Lys05 Induces the Early-Stage Autophagy but Subsequently Leads to Its Inhibition Resulting in Lysosome Accumulation

To further study the impact of autophagy inhibitors and IR on lysosomes, we used fluorescence microscopy focused on lysosome visualization and flow cytometry for quantification of changes in their fluorescence intensity. In both cases, we used a fluorescence dye Lysosensor Green DND-189 (LSG). LSG is a weak base that accumulates in acidic organelles. It can be used to measure the pH of acidic organelles—such as lysosomes—as it becomes more fluorescent in acidic environments.

**Figure 4:**
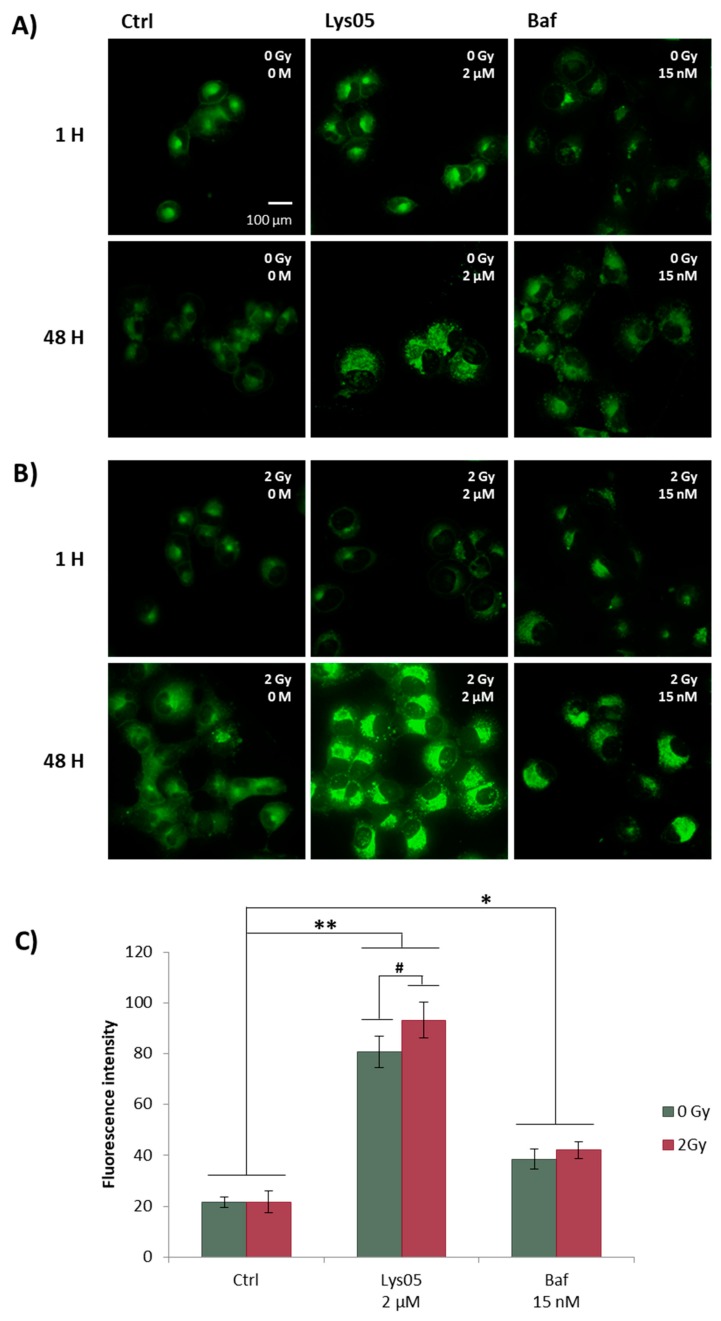
Administration of Lys05 leads to the accumulation of lysosomes. The H1299 cells were treated either by IR or the inhibitor alone or by their combination. Lys05 in 2 µM and Baf in 15 nM concentrations were added to the cells one hour prior to irradiation (2 Gy). For the sake of visualization, the cells were stained with the green dye—LysoSensor Green DND-189. (**A**) Non-irradiated H1299 cells were imaged by fluorescence microscopy at intervals of one and 48 h after the treatment. (**B**) Irradiated H1299 cells were imaged by fluorescence microscopy at intervals of one and 48 h after irradiation. (**C**) The intensity of fluorescence was measured by flow cytometry 48 h after irradiation only. The intensity plot displays average values ±SD from one experiment performed in triplicate * Significant difference compared to control (*p*-value ≤ 0.05). ** Significant difference compared to control (*p*-value ≤ 0.01). # Significant difference compared to non-irradiated group (*p*-value ≤ 0.05).

We studied H1299 cells both one and 48 h after irradiation (2 Gy) pre-treated by Lys05 (2 µM) and Baf (15 nM) one hour prior to IR. Baf was selected as a control in this method because of its mechanism of action similar to Lys05—blockade of autophagosome-lysosome fusion. We presumed that using Baf as a control would enable comparison of the characteristics and intensity of the inhibition (rate of the autophagosome or lysosome accumulation). One hour after irradiation, we did not observe any changes in fluorescence intensity, cell shape, or lysosome number. However, 48 h after irradiation, we observed the increased granularity of cells followed by the increased fluorescence intensity and changes in cell size—cell enlargement, which could be caused by the accumulation of lysosomes. Similar results were obtained by experiments with Baf (Figure 4A,B).

In order to quantify changes in fluorescence intensity, we performed flow cytometry determination with the same treatment scheme as used for fluorescence microscopy. Since no changes in fluorescence intensity were observed in the group visualized one hour after irradiation, we quantified the fluorescence intensity 48 h after irradiation only. The results from flow cytometry correlate with those from fluorescence microscopy, demonstrating a significant increase in fluorescence intensity in the cells treated both with Lys05 and Baf (Figure 4C). We speculate that such a substantial increase in fluorescence intensity might be the result of two subsequent events: (i) Activation of autophagy by the inhibitor in the early stage, and (ii) actual inhibition of autophagy due to blockade of autophagosome-lysosome fusion in the late stage of the autophagic process.

### 2.6. Lys05 Potentiates Autophagy Inhibition in H1299 Cells via Accumulation of Autophagosomes

**Figure 5:**
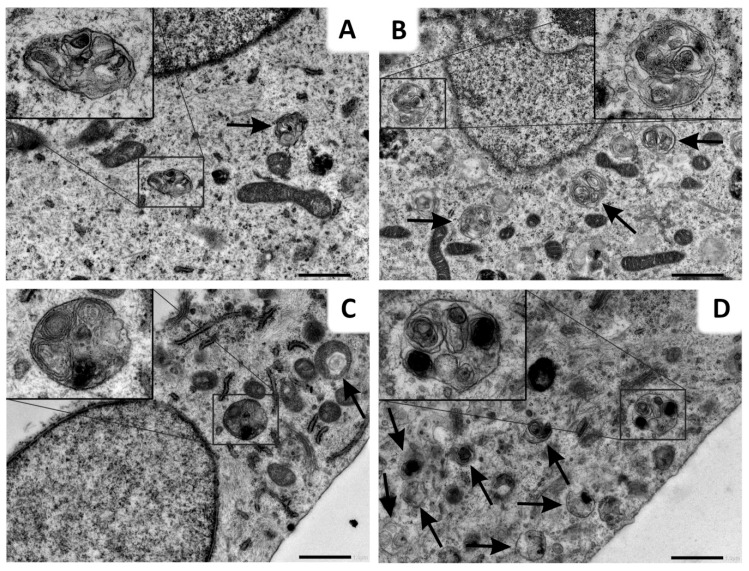
Administration of Lys05 leads to the accumulation of autophagosomes. (**A**) Some of the control group cells contained autophagosomes in their cytoplasm. In comparison with cells of other experimental groups, autophagosomes of the control cells were apparently smaller and less numerous. (**B**) Lys05-treated cells (2 µM) contained medium-sized and frequent autophagosomes. (**C**) In irradiated cells (2 Gy), large autophagosomes were often observed in their cytoplasm. (**D**) All irradiated and Lys05-treated cells (2 Gy + 2 µM) contained numerous autophagosomes of various sizes in their cytoplasm. Black arrows indicate autophagosomes. Scale bar (**A**–**D**) 1 µm (mag. 8000×); insert mag. (**A**) 19700×, (**B**) 17000×, (**C**,**D**) 17600×.

Consistently with fluorescence microscopy and flow cytometry, transmission electron microscopy (TEM) was used for ultrastructural visualization. Autophagy suppression induced by the inhibitor in the late stage of the autophagic process resulted in autophagy vacuole accumulation. Autophagy was inhibited in cells treated by Lys05 alone, which is evidenced by the increased number of autophagic vesicles. The cells irradiated by a dose of 2 Gy also showed a higher number of vesicles in comparison to the control group. Importantly, the combination of Lys05 and IR resulted in a substantial increase in the number of autophagic vacuoles in H1299 cells, indicating the efficient inhibition of autophagy (Figure 5).

**Figure 6:**
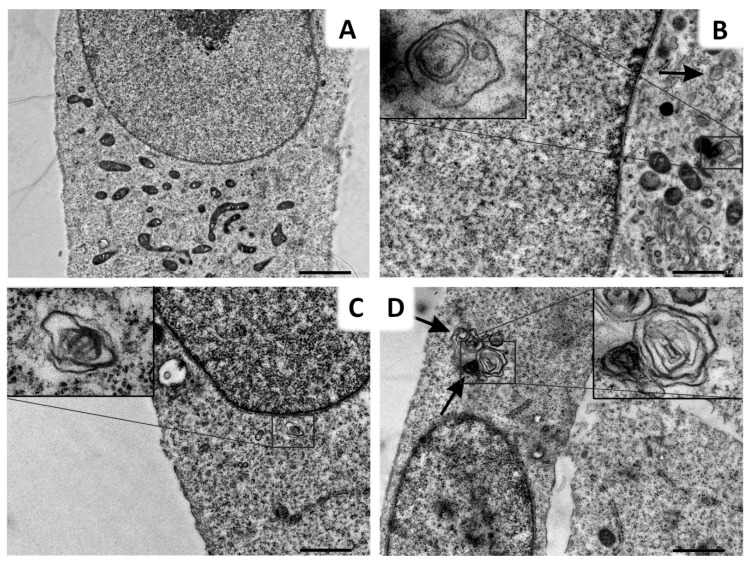
Administration of Baf leads to the accumulation of autophagosomes. (**A**) Several control group cells contained structures resembling autophagosomes, but they lacked some characteristic features—they were not double-membraned. (**B**) Baf-treated cells (15 nM) contained medium-sized autophagosomes (arrow). (**C**) In solely irradiated cells (2 Gy) autophagosomes were formed as well. (**D**) Almost all irradiated and Baf treated cells (2 Gy + 15 nM) contained numerous autophagosomes (arrows) in their cytoplasm. Scale bar (**A**) 2 µm (mag. 4 000×); (**B**–**D**) 1 µm (mag. 8000×); insert mag. (**B**) 32000×, (**C**) 31600× and (**D**) 21000×.

In order to validate our data, we compared the effect of Lys05 with Baf treatment. Similarly to fluorescence microscopy, we aimed to compare the nature of autophagosome–lysosome blockade, therefore we selected an inhibitor with a similar mechanism of action as Lys05. Initially, we found out that several control group cells contained structures resembling autophagosomes. They lacked some characteristic features—they were not double-membraned. On the other hand, Baf-treated cells contained medium-sized autophagosomes in their cytoplasm as well as irradiated-only cells. Similarly to Lys05, the combination of the inhibitor and IR led to the intensive formation of autophagosomes, as the majority of the cells contained more autophagosomes than after IR- or Baf-treatment only (Figure 6).

## 3. Discussion

The majority of patients with NSCLC are treated by RT. Autophagy is believed to be a radioprotective mechanism in cancer cells. Thus, its inhibition may render cells more vulnerable, thereby increasing the efficiency of RT. We employed radioresistant human non-small cell lung carcinoma cells (H1299, p53-negative) in order to examine their response to IR and combined treatment with the specific autophagy inhibitor Lys05.

Autophagy inhibitors have been addressed in a plethora of studies with the aim of the radiosensitization of cancer cells. For example, CQ derivatives are commonly-used inhibitors of autophagy which are already approved for clinical trials focusing on cancer therapy [17]. Regarding these studies, Toulany et al. [18], as well as Karagounis et al. [19], reported the radiosensitizing effect of CQ on lung cancer cells as a result of autophagy blockade. Even though both CQ and HCQ can effectively inhibit autophagy, the doses necessary for the appropriate effect in vitro are not consistently achievable in patients, and there is an identified need for new inhibitors with better physicochemical and pharmacokinetic properties. The autophagy inhibitor Lys05, a derivative of CQ, was described by Amaravadi et al. in 2012 [20]. Lys05 can accumulate inside the lysosomes more potently than HCQ and is, therefore, a promising newly-developed autophagy inhibitor.

To study the radiosensitizing effect of Lys05, we applied real-time monitoring of H1299 cell proliferation by which we detected a concentration-dependent decrease in cell proliferation. According to our data, we argue that such treatment might contribute to suppressed autophagy that serves as a promoter of carcinogenesis in advanced tumors, providing nutrients for higher metabolic requirements [4,21]. Moreover, a drop in cell proliferation after combined treatment by IR suggests that pre-incubation with Lys05 leads to the radiosensitization of H1299 cells.

In regards to molecular mechanisms of Lys05 action, we examined the level of the key regulatory proteins of the autophagic process as well as gene expression of related genes: *SQSTM1* and *BNIP3*. Since protein p62/SQSTM1 accumulates inside the cells when autophagy is suppressed, and it is degraded during the autophagic process, it was established as a marker of autophagy [15]. It has been generally accepted that IR induces autophagy in radioresistant H1299 cells [22] and in several other cell lines [23]. In this respect, we observed the rather unchanged levels of protein p62/SQSTM1 and its coding gene *SQSTM1* in solely-irradiated H1299 cells after 48 h. On the other side, we found an elevated level of p62/SQSTM1 together with increased gene expression of *SQSTM1* 48 h after IR combined with pre-treatment by Lys05. These findings are consistent with the study of Koukourakis et al., who similarly described an unchanged level of p62/SQSTM1 in the solely-irradiated and elevated level of p62/SQSTM1 in Baf-pre-treated radioresistant PC3 prostate cancer cells [21].

Furthermore, in ongoing autophagy, BNIP3 interacts with LC3 to recycle endoplasmic reticulum and mitochondria. When inactive BNIP3 is activated, LC3 binds to the LC3-interacting region motif on BNIP3 and facilitates the formation of an autophagosome [24]. Since activation of BNIP3 is a pro-autophagic mechanism [16], downregulation of the expression of its coding gene *BNIP3* may indicate the inhibition of autophagy [25]. Besides, LC3 is cleaved to LC3-I (cytosolic form) and LC3-II (membrane-associated form) during autophagy. Thus, detectable LC3 cleavage is generally considered as a marker of ongoing autophagic flux. Physiologically, LC3-II is in later stages of autophagy degraded by lysosomal hydrolases along with intra-autophagosomal content resulting in complete LC3 disappearance [14,26]. However, using of specific autophagy inhibitors, e.g., Baf [6,27], might lead to a late-stage increase in LC3-II, consistent with our data, that suggests either the enhanced autophagosome synthesis or reduced autophagosome recycling [28,29].

Moreover, in terms of autophagy inhibition, interpreting p62/SQSTM1 level or LC3II/I ratio separately is discouraged in favor of the conclusions provided by their mutual interpretation. In this respect, Mizushima and Yoshimori demonstrated that increased levels of p62/SQSTM1 together with elevated LC3II/I ratio indicate rather the inhibition of autophagic process than its activation [14]. On this basis, we came to an assumption of the late-stage autophagy inhibition because of the blockade of autophagosome-lysosome fusion.

Apparently, there are two traceable hallmarks that accompany the late-stage autophagy inhibition: (i) accumulation of autophagosomes and (ii) associated accumulation of lysosomes. According to our TEM data, increased accumulation of autophagic vacuoles was observed both in the Baf- and Lys05-treated cells. Importantly, the cells treated by a combination of Lys05 with IR exhibited substantial accumulation of autophagic vacuoles. This is consistent with the study by Makowska et al. who observed elevated levels of autophagosomes after co-treatment of nasopharyngeal carcinoma by CQ and IR [30].

Using fluorescence microscopy, we detected a significant increase in LSG fluorescence intensity (in parallel quantified by flow cytometry) in cells treated by either IR alone or in combination with the inhibitors of autophagy. We assume that such an increase might be the result of lysosome accumulation caused by the blockade of autophagosome–lysosome fusion. These results are in correlation with studies by Lu et al. [31] and Chikte et al. [32], who used CQ as an autophagy inhibitor. In both studies, the time- and dose-dependent increase in fluorescence intensity was observed. According to Lu et al. an increase in LSG staining intensity could be attributed to the elevated lysosome volume [31]. Similarly, Chikte et al. speculated that CQ induced the autophagic process in early stages but consequently, as the process progressed, it inhibited the autophagosome–lysosome fusion resulting in an incomplete autophagic flux [32].

Lys05 seems to be a promising autophagy inhibitor. Several studies engaging with its effects on tumor cells have been published lately. For example, Gade et al. used Lys05 for the treatment of rat hepatocellular carcinoma in association with transarterial embolization [33]. Ndoye et al. studied the link between the Wnt5A pathway and autophagy in melanoma cells [34]. DeVorkin et al. examined hypoxia-induced autophagy inhibition using Lys05 in combination with sunitinib in clear cell ovarian carcinoma demonstrating the remarkable chemosensitizing effect of the autophagy inhibitor—Lys05 [35].

## 4. Materials and Methods

### 4.1. Cell Cultures and Cultivation

Non-small cell lung carcinoma cells (H1299) were obtained from the American Type Culture Collections (Manassas, VA, USA). The cells were cultured at 37 °C in a humidified incubator under a controlled 5% CO_2_ atmosphere and maintained in RPMI medium 1640 (Gibco, Paisley, UK) supplemented with 10% fetal bovine serum, 150 UI/mL penicillin, and 50 mg/mL streptomycin (all from Sigma-Aldrich, St. Louis, MO, USA). The culture was divided twice per week by dilution to a concentration of 2 × 10^5^ cells/10 mL. The cell line in the maximal range of up to 15 passages was used for this study.

### 4.2. Cell Treatment

The cells were treated by autophagy inhibitors Lys05 (a kind gift from Dr. Amaravadi, Pennsylvania University, Philadelphia, PA, USA) and Spautin-1 (Calbiochem, San Diego, CA, USA) in various concentrations of 2, 5, and 10 μM and Baf in 15 nM concentration. Inhibitors were added to the cells 1 h prior to irradiation. The cells in either flask, tube, or 6-well plate, were irradiated using a ^60^Co gamma-ray source with a dose rate of 0.44 Gy/min. After irradiation, the cells were transferred back into an incubator and cultivated further for the prescribed time according to the particular experiment.

### 4.3. Cell Growth Real-Time Monitoring

The xCELLigence system was used according to the manufacturer’s instructions (ACEA Biosciences, Inc.; San Diego, CA, USA). First, 50 µL of a growth medium was added to each well, and the background impedance was measured. One hundred microliter of cell suspension (a total number of 3 × 10^3^ per well) were then added, and the plate was left at room temperature for 30 min to allow the cells to attach homogeneously. The cells were treated by either IR or inhibitor alone, or by their combination. Various concentrations of both Spautin-1 and Lys05 (2, 5, and 10 μM) were added into the cell culture 1 h prior to irradiation (2, 4, and 8 Gy). The cell proliferation was measured and recorded continuously every 30 min for 5 days. Statistical analysis was performed by an independent two-sample t-test with unequal variances and a critical *p*-value equal to 0.05.

### 4.4. Clonogenic Assay

The cells were trypsinized, re-plated in 6-well plates, treated by Lys05 (2 µM) 1 h prior to irradiation (2, 4, 6, and 8 Gy) and then left to form colonies. After a 10-day incubation period (37 °C, 5% CO_2_), colonies were stained using 1% crystal violet in methanol and counted by eye with a cut-off of 50 viable cells.

### 4.5. The Combination Index and a Time-Dependent Combined Toxicity Level Calculations

The real-time monitoring data were used to calculate both the CI and a time-dependent combined toxicity level. Median-effect computer software CompuSyn for Windows (ComboSyn, Inc., available here: http://www.combosyn.com) set up by Dr. Dorothy Chou was used to generate the combination index values. The extent of synergy/antagonism was evaluated according to the CI value description published by Hernandez et al. where CI values under the 0.9 indicate synergism, CI values between 0.9 and 1.2 indicate additive effect, and CI values higher than 1.2 indicate antagonism (full CI value description table available here: [13]).

A time-dependent combined toxicity levels were calculated based on these equations:

(1) $$T_{L+IR}=\frac{\left(T_{L}+T_{IR}\right)}{2*T_{Ctrl}}$$

(2) $$T_{LIR}=\frac{\left(T_{LIR\_comb}\right)}{T_{Ctrl}}$$

(3) $$T_{S+IR}=\frac{\left(T_{S}+T_{IR}\right)}{2*T_{Ctrl}}$$

(4) $$T_{SIR}=\frac{\left(T_{SIR\_comb}\right)}{T_{Ctrl}}$$

where T, in general, represents the antiproliferative/toxic effects of Lys05, Spautin-1, IR, or their combination. Following, T_L+IR_ represents the antiproliferative/toxic effect of the sum of Lys05 and IR when acting as single agents, T_LIR_ represents the antiproliferative/toxic effect of the combination of Lys05 and IR, T_S+IR_ represents the antiproliferative/toxic effect of the sum of Spautin-1 and IR when acting as single agents, and T_SIR_ represents the antiproliferative/toxic effect of the combination of Spautin-1 and IR.

### 4.6. Western Blot

The cells were harvested at different time intervals, and proteins were extracted using lysis buffer (137 mM NaCl; 10% glycerol; 1% n-octyl-β-glucopyranoside; 50 mM NaF; 20 mM Tris, pH 8; 1 mM Na_3_VO_4_) supplemented by cocktail protease inhibitor (Complete Mini, Roche, Basel, CHN). Equivalent amounts of samples (30 μg) were mixed with sample loading buffer and heated at 95 °C for 5 min. Proteins were separated on 10% gel by SDS-PAGE (200 V, 40 min) and subsequently transferred onto a PVDF membrane (100 V, 120 min). The membrane was blocked using 5% non-fat dry milk in TBST at room temperature (RT) for 1 h. After blocking, primary antibodies (p62/SQSTM1 Antibody 1:1000; LC3B Antibody 1:1000; p-PRAS40 (Thr246) Antibody 1:1000; Cell Signaling Technology, Danvers, MA, USA) were added and incubated overnight on a shaker at 4 °C. Afterward, the membrane was rinsed with TBST (3 × 10 min) and incubated with peroxidase-labeled secondary antibody (Dako, Glostrup, DEN) at RT for 1 h. The membrane was developed using ECL reagent (BM Chemiluminescence—POD, Roche, Manheim, GER). Representative blots from three independent experiments are shown.

### 4.7. Real-Time Quantitative Reverse Transcription PCR

The cells were lysed using the RLT buffer (QIAGEN, Hilden, Germany) with the addition of 1% β-mercaptoethanol 1, 24, and 48 h after irradiation. Then, RNA was isolated from the lysates using the RNeasy lipid tissue mini kit (QIAGEN) and QIAcube (QIAGEN) according to the manufacturer’s instructions. The quality and concentration of eluted RNA was assessed spectrophotometrically on NanoDrop One Microvolume UV-Vis Spectrophotometer (Thermo Fisher Scientific, Waltham, MA, USA). Two-step qRT-PCR was used to analyze the changes in gene expression. In the first step, RNA (1 µg) in a concentration of 100 ng/µL was transcribed into cDNA using the high capacity cDNA reverse transcription kit (Thermo Fisher Scientific) according to the manufacturer’s instructions. In the second step, each qRT-PCR reaction was prepared according to the manufacturer’s instructions in duplicate containing TaqMan fast advanced master mix (Life Technologies, Thermo Fisher Scientific), *TBP* (Hs00427620_m1), *SQSTM1* (Hs00177654_m1), *BNIP3* (Hs00969291_m1) probes and 10 ng (1 ng/µL) of cDNA template. The qRT-PCR analyses were performed using the fast-cycling profile of StepOnePlus real-time PCR system and StepOnePlus Software (Life Technologies, Thermo Fisher Scientific). Obtained data were analyzed by the Livak method [36] using *TBP* as a reference gene. Statistical analysis was performed by an independent two-sample t-test with unequal variances and a critical *p*-value equal to 0.05.

### 4.8. Transmission Electron Microscopy

The cells were harvested 45 h after irradiation. Then, 3% glutaraldehyde (in 0.1 M cacodylate buffer, pH 7.2; Sigma) was used for cell fixation and the cells were fixed for 5 min at 37 °C and then for 3 h at room temperature. Subsequently, the cells were washed in cacodylate buffer (0.1 M, pH 7.2) and post-fixed in 1% OsO_4_ (in 0.1 M cacodylate buffer, pH 7.2; Sigma) for 1 h at room temperature. After the rinsing procedure, the cells were dehydrated in graded alcohols (50%, 75%, 96%, and 100%), clarified in propylene oxide and embedded in a mixture of Epon 812 and Durcupan (Sigma; polymerization for 3 days at 60 °C). Toluidine blue was used to stain the semithin sections. Ultrathin sections were cut on Ultrotome Nova (LKB, Sweden). These sections were then collected onto formvar carbon-coated copper grids, counterstained with uranyl acetate and lead citrate and examined under JEOL JEM-1400Plus transmission electron microscope (at 120 kV JEOL, Japan). The images were taken with the integrated 8Mpix CCD camera and processed further using software TEM Center (Ver. 1.7.3.1537, JEOL, Japan).

### 4.9. Fluorescence Microscopy

One and 48 h after irradiation, the cultivation medium was replaced by 1 µM LysoSensor Green DND-189 in a fresh medium, and the cells were then incubated for an additional 30 min. After incubation, the cells were washed twice with PBS and examined immediately under the Olympus BX51 microscope (Olympus, Tokyo, Japan) with a green excitation fluorescence filter. The images were captured by the integrated Olympus DP73 camera (Olympus, Tokyo, Japan) and processed using microscope imaging software cellSens (Entry, Olympus, Tokyo, Japan).

### 4.10. Flow Cytometry

One and 48 h after irradiation, the cultivation medium was replaced by 1 µM LysoSensor Green DND-189 in a fresh medium, and the cells were then incubated for an additional 30 min. After incubation, the cells were washed twice with PBS and scraped off with a scraper. The cells were analyzed immediately using CyAn ADP Flow Cytometer (Beckman Coulter, Inc., Brea, CA, USA) and the obtained data were then processed by the flow cytometry analysis software Summit 4.2 (Beckman Coulter, Inc., Brea, CA, USA). Statistical analysis was performed by an independent two-sample t-test with unequal variances and critical *p*-values equal to 0.05 and 0.01, respectively.

## 5. Conclusions

Taken together, co-treatment of H1299 cells by Lys05 and IR caused significant autophagy inhibition compared to the control and the solely-irradiated group. According to our data, autophagy is blocked by Lys05 in the later stage of the autophagic process, autophagosome–lysosome fusion.

Concerning autophagy, the current literature is conflicting. As Karagounis et al. concluded that the impact of IR on autophagy is complex and varies not only with cell type and radiation dose but is influenced by many other factors [19]. Although some groups reported IR-induced autophagy, others including us observed autophagy that was inhibited (possibly because of different experimental setup). Obviously, general conclusions and implications for therapy are limited. A crucial question is whether it is more beneficial to support autophagy inhibition or induction in order to diminish most of the cancer cells.

While several papers already covered the chemo-sensitizing effect of Lys05, more has to be learned about its application together with IR. Overall, this study contributes to the limited knowledge about novel autophagy inhibitor—Lys05—presenting the first study so far dedicated to combination with radiation. Our data indicate that the autophagy inhibitors can be used as an effective tool for the radiosensitization of H1299 cells, and it is tempting to conclude that it might provide a promising approach for lung cancer therapeutic strategy.

## Figures and Tables

**Table 1:** Combination index values for the combination of autophagy inhibitors Lys05 and Spautin-1 with IR. Data are of values from Figure 2. Fa = Fraction affected/cytotoxicity and CI = combination index.

IR Dose [Gy]	Dose of Lys05 [µM]	Fa	CI	IR Dose [Gy]	Dose of Spautin-1 [µM]	Fa	CI
2	2	0.39	1.02	2	2	0.43	1.04
2	5	0.47	0.94	2	5	0.39	1.91
2	10	0.52	0.97	2	10	0.65	0.83
4	2	0.52	1.23	4	2	0.65	0.92
4	5	0.65	0.93	4	5	0.63	1.14
4	10	0.58	1.27	4	10	0.74	0.89
8	2	0.77	1.23	8	2	0.85	0.93
8	5	0.82	1.02	8	5	0.82	1.07
8	10	0.78	1.20	8	10	0.85	0.96

## Supplementary Materials

Supplementary materials can be found at https://www.mdpi.com/1422-0067/20/23/5881/s1.

## Abbreviations

Baf	Bafilomycin A1
BNIP3	Bcl2 interacting protein 3
CI	Combination Index
CQ	Chloroquine
H1299	Non-small cell lung carcinoma cell line
HCQ	Hydroxychloroquine
IR	Ionizing radiation
LC3	Microtubule-associated protein 1A/1B light chain 3
LSG	Lysosensor Green DND-189
Lys05	Coding name for an autophagy inhibitor
mTOR	Mammalian target of rapamycin
NSCLC	Non-small cell lung carcinoma
p53	Cellular tumor antigen 53
p62	Ubiquitin-binding protein p62
PRAS40	Proline-rich Akt substrate of 40 kDa
RT	Radiation therapy
SQSTM1	Sequestosome-1
TEM	Transmission electron microscopy
ULK-1	Unc-51 like autophagy activating kinase 1
