# Supplementary material for: A Potent Autophagy Inhibitor (Lys05) Enhances the Impact of Ionizing Radiation on Human Lung Cancer Cells H1299
Source: Int J Mol Sci. 2019 Nov 23;20(23):5881. doi: 10.3390/ijms20235881 (PMC6928878; doi:10.3390/ijms20235881)
Supplement: Supplementary file 1 [file ijms-20-05881-s001.zip › Supplementary Figures.pdf]

Figure S1. Autophagy inhibitors caused decline in H1299 proliferation - full interpretation of the real-time monitoring data

Continual cell proliferation monitoring by xCELLigence system was used in order to evaluate an effect of autophagy inhibitors and IR on H1299 cells. The cells were treated either by autophagy inhibitors Lys05 or Spautin-1 alone or by IR alone or by a combination of inhibitor and IR. Both inhibitors in final concentrations of 2, 5, and 10 μM were added to the cells 1 hour prior to irradiation (2, 4, and 8 Gy). Cell proliferation index was measured by xCELLigence system each 30 minutes for 5 days.

Creation of names in the chart legend:

X(first letter of the inhibitor name)-Y(concentration of the inhibitor) + Z(irradiation dose)

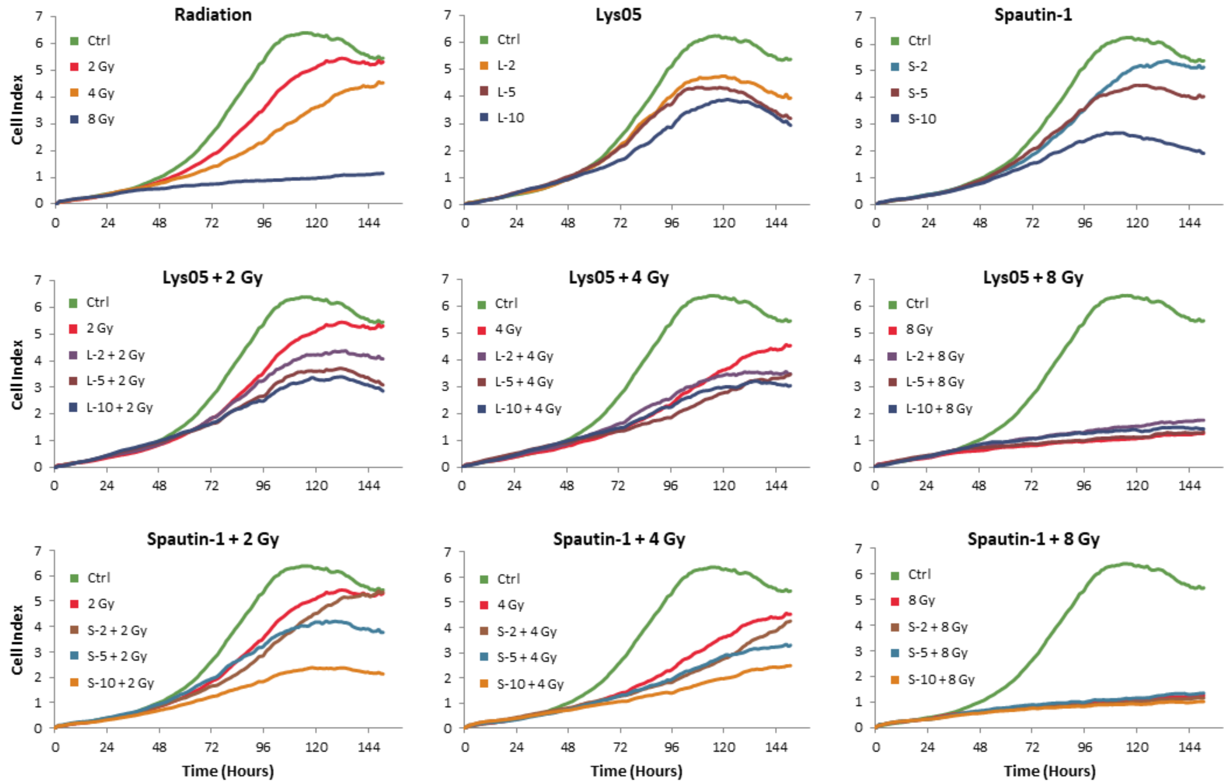

Figure S2. Administration of Lys05 blocked autophagosome degradation

The original unmodified scans of Western Blot analysis are shown.

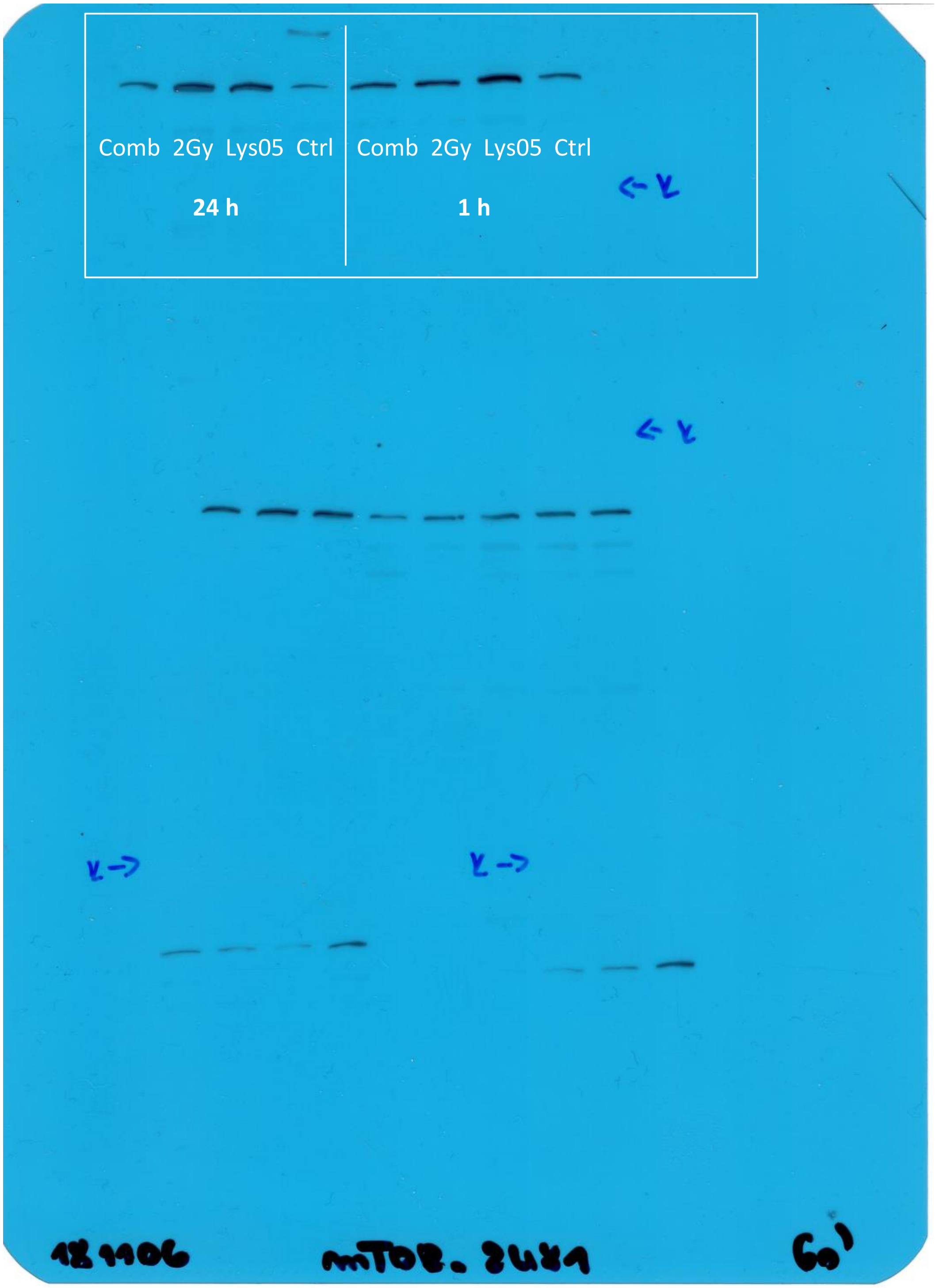

1. mTOR_Ser2481; 1 and 24 h

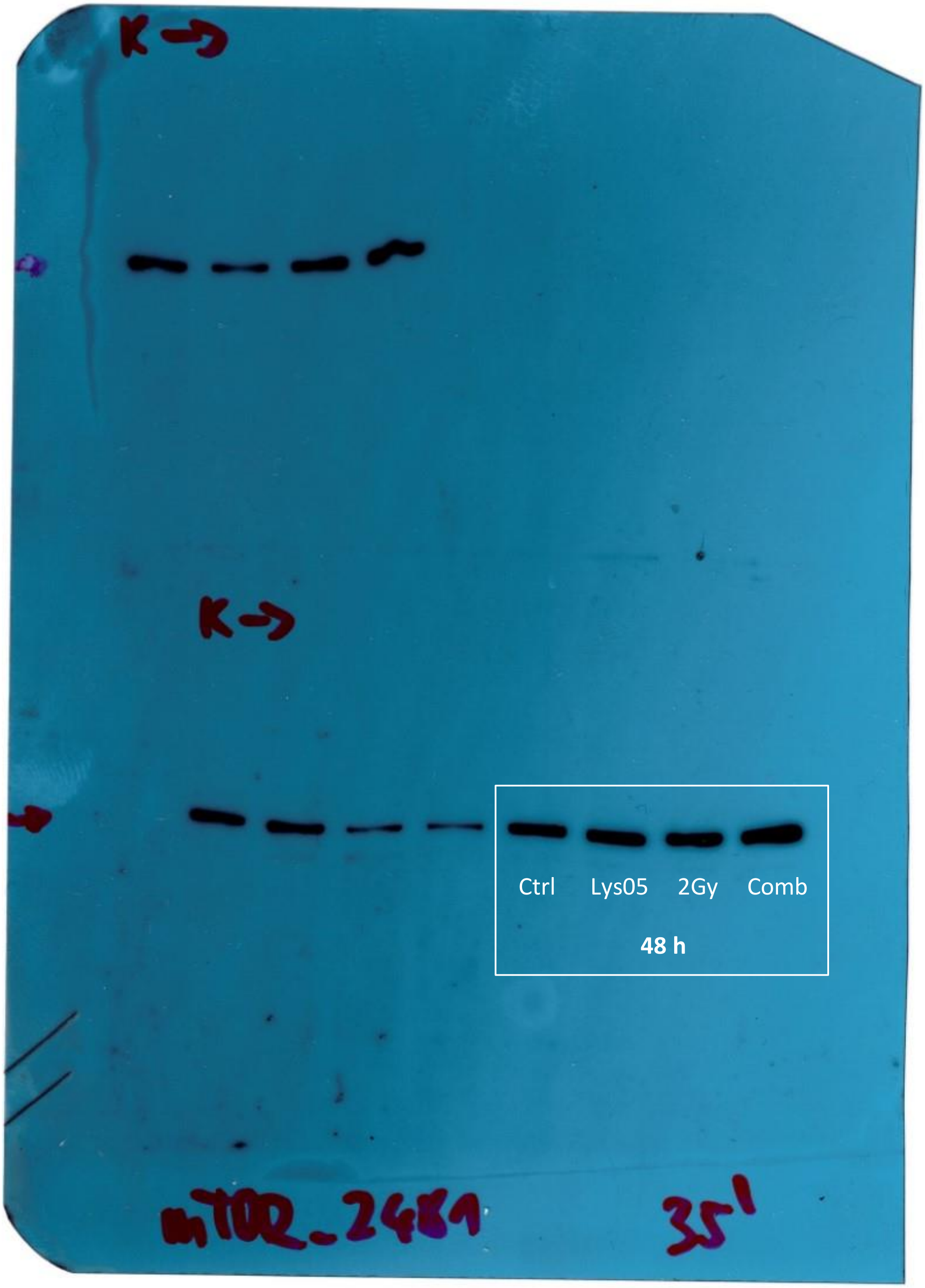

1. mTOR_Ser2481; 48 h

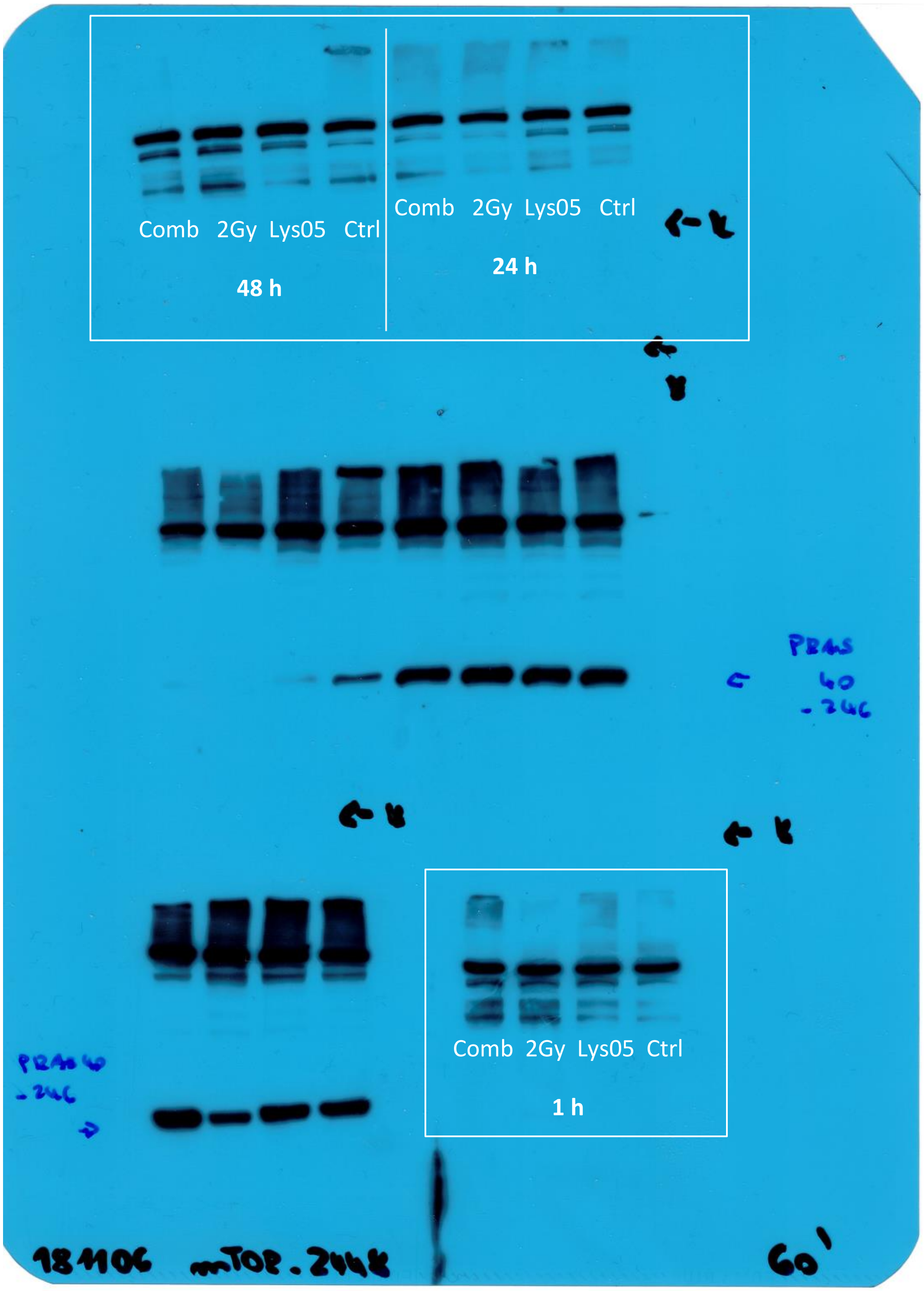

2. mTOR_Ser2448

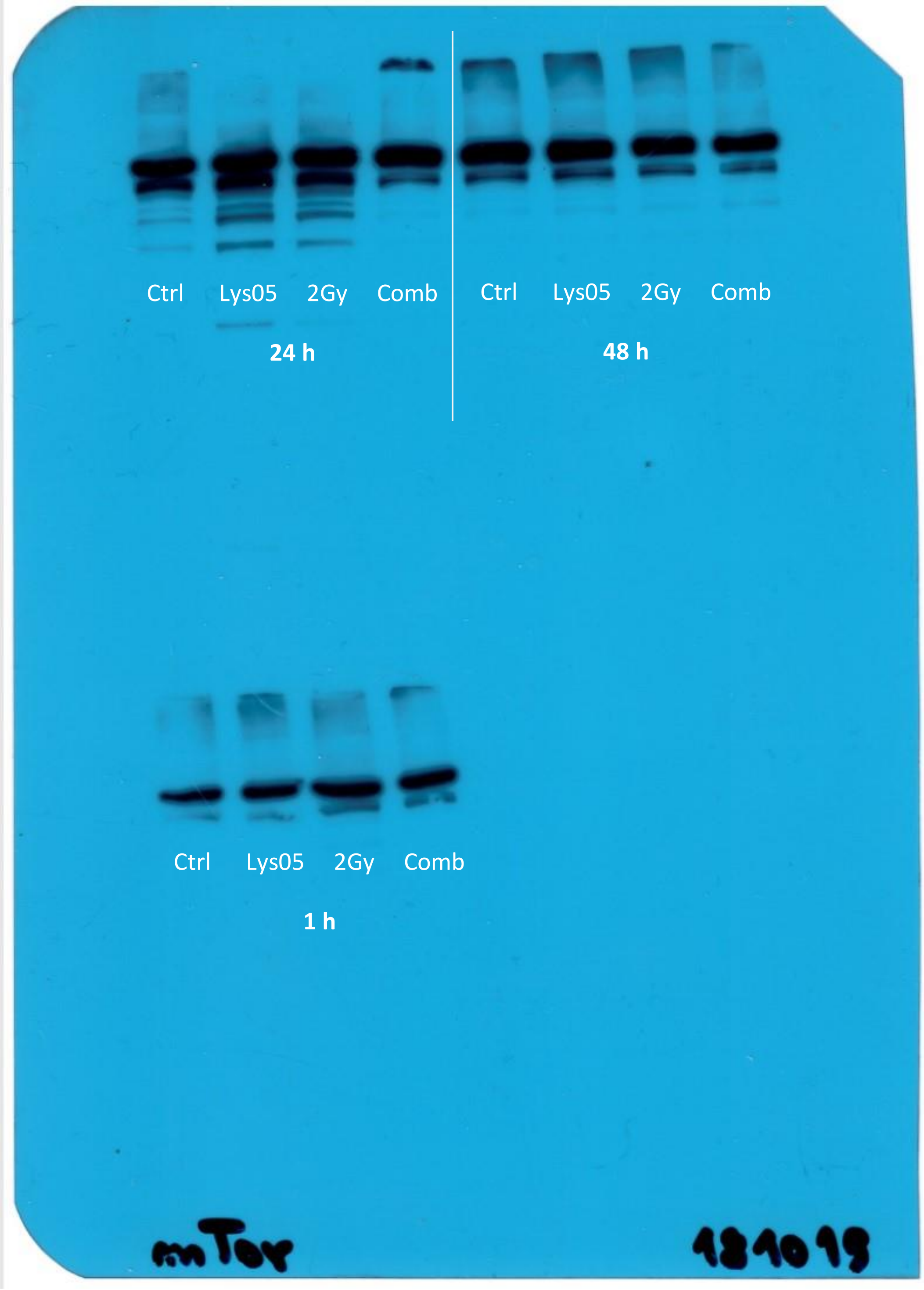
Ctrl
Lys05
2Gy
Comb
24 h
Ctrl
Lys05
2Gy
Comb
48 h
Ctrl
Lys05
2Gy
Comb
1 h
mTor
181018

3. mTOR

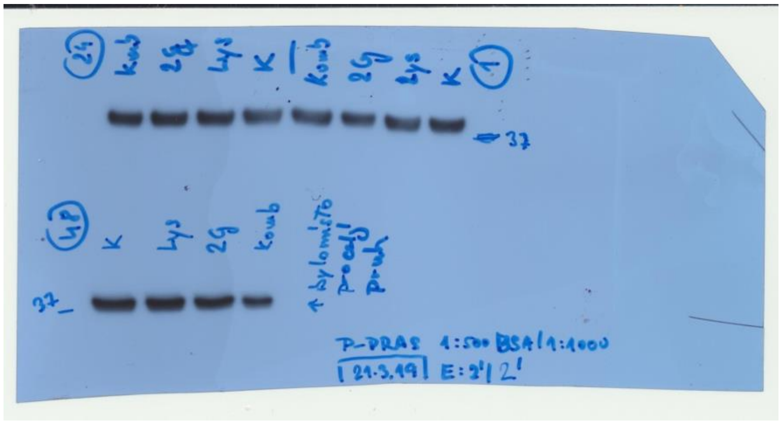

4. PRAS40_Thr246

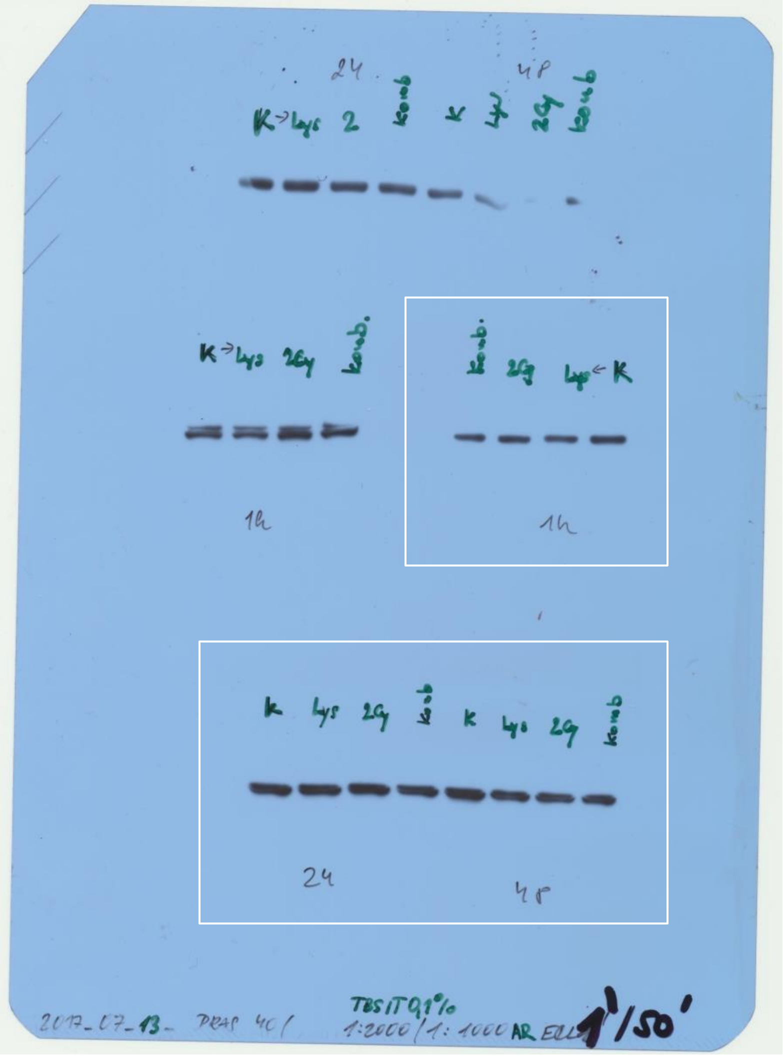
24
48
K→Lys
2Gy
komb
1h
2017_07_13_ PRAS 40/
TBS/T 0,1%
1:2000/1:1000 AR ECL
1'/50'

5. PRAS40

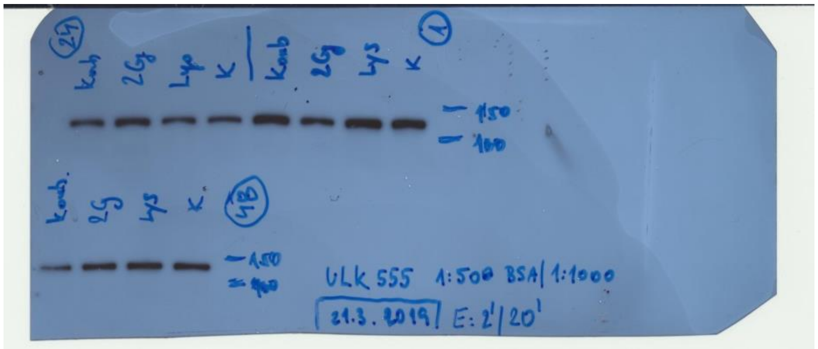

6. ULK-1_Ser555

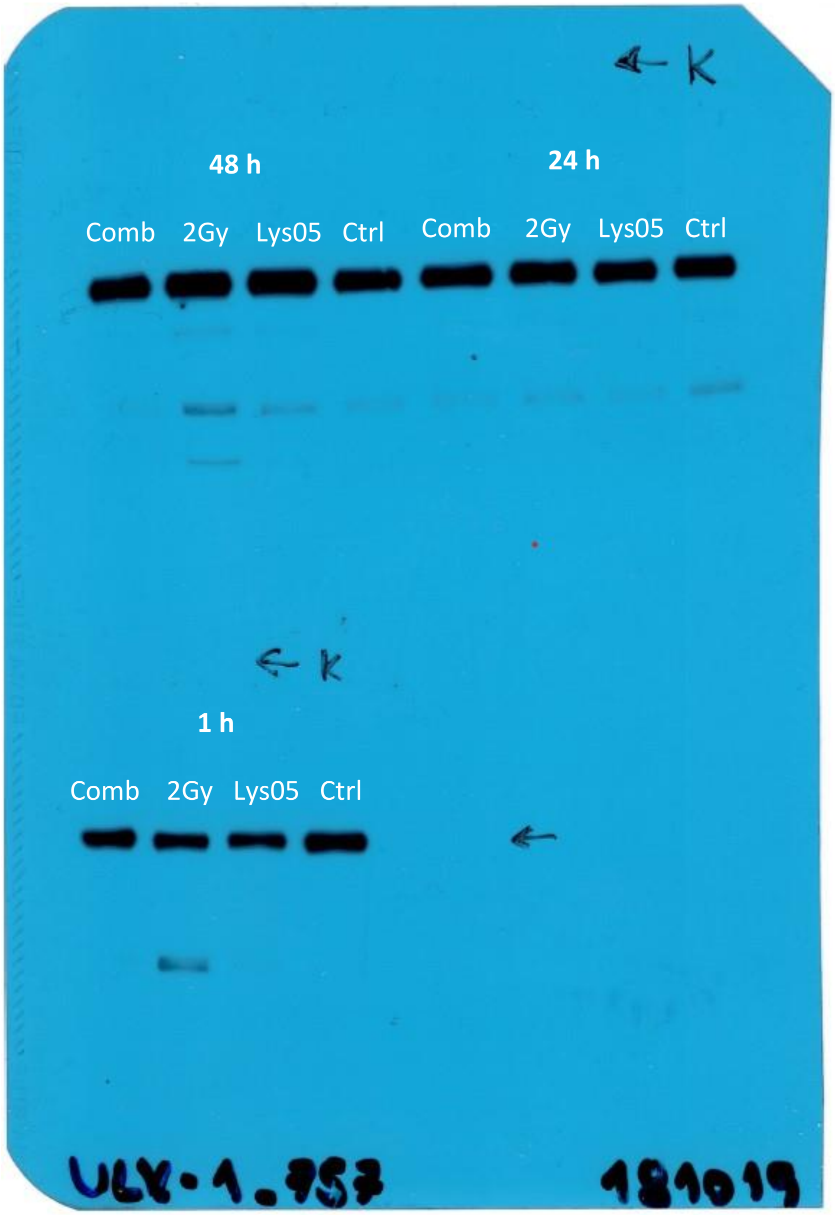

7. ULK-1_Ser757

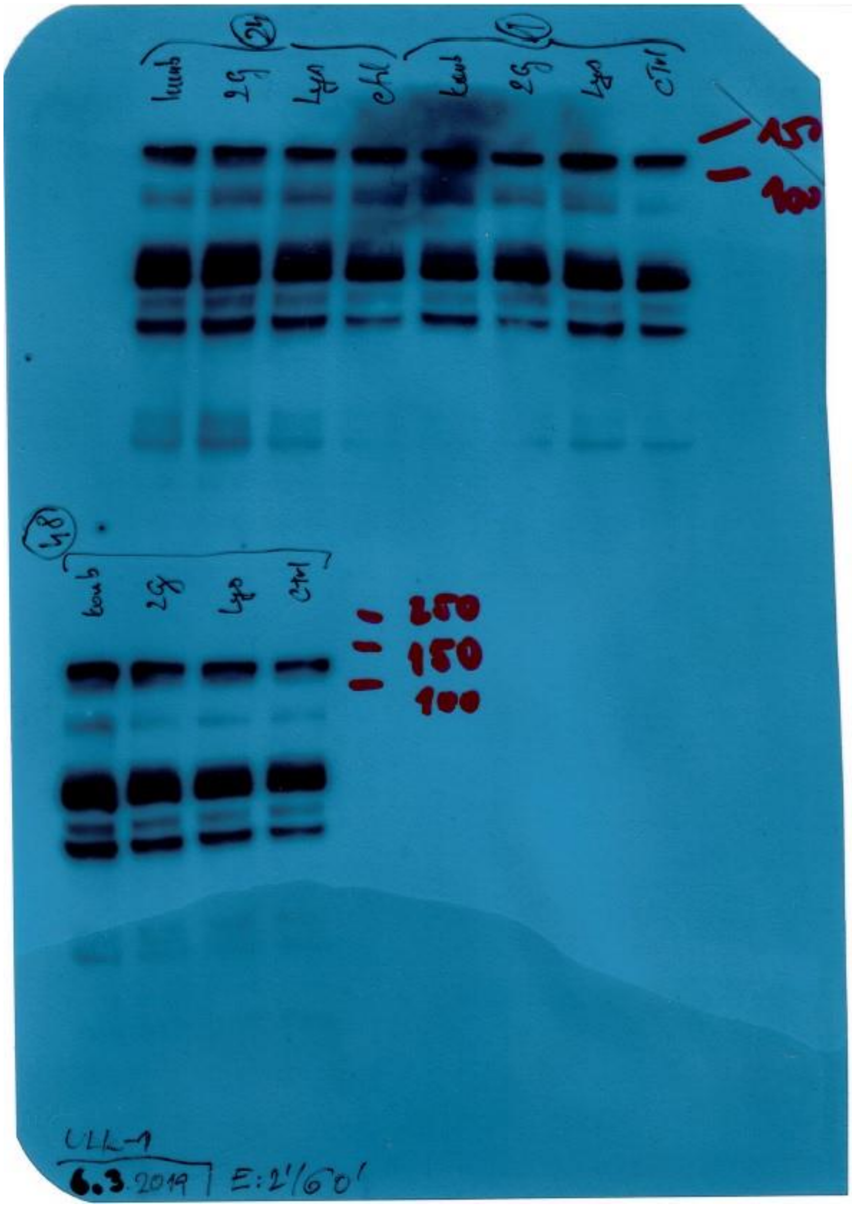

24
kaub
2G
Lyo
Ctrl
kaub
2G
Lyo
Ctrl
150
100
48
kaub
2G
Lyo
Ctrl
250
150
100
ULK-1
6.3.2019
E: 2'/60'

8. ULK-1

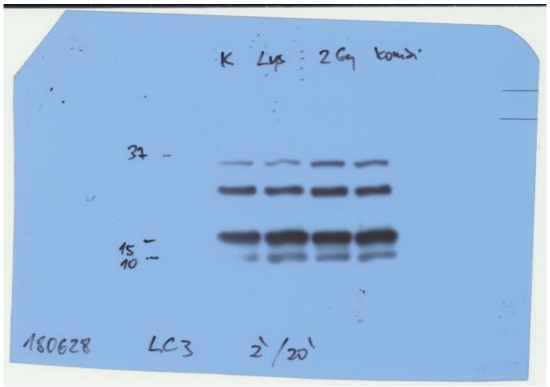

9. LC3-I/II; 1 h

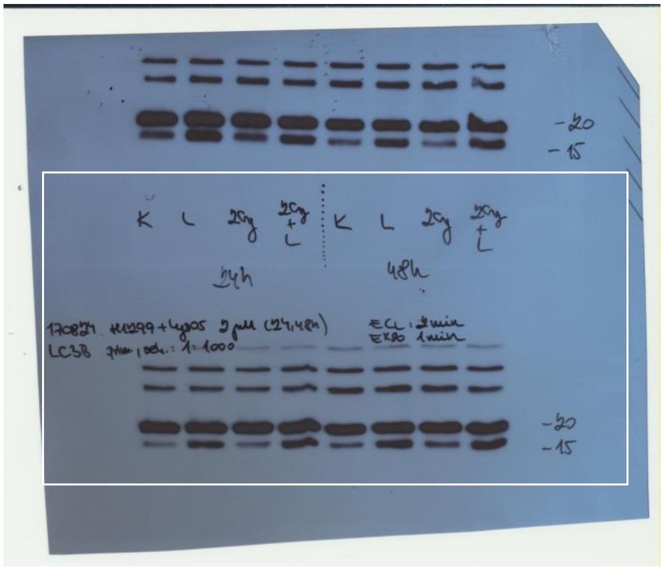

9. LC3-I/II; 24 and 48 h

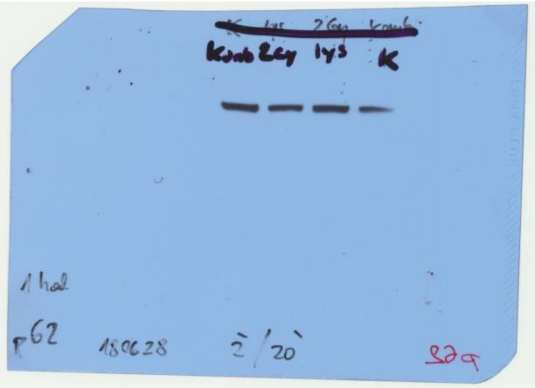

10. p62/SQSTM1; 1 h

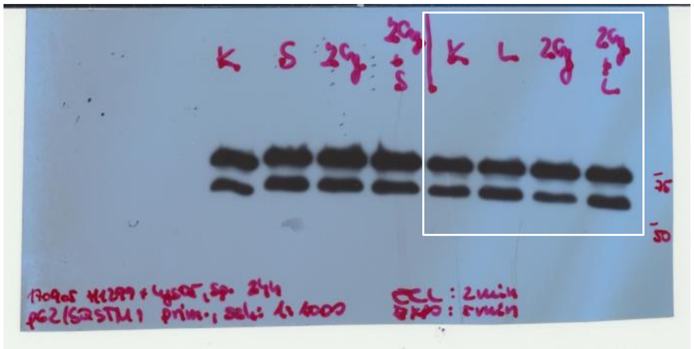

10. p62/SQSTM1; 24 h

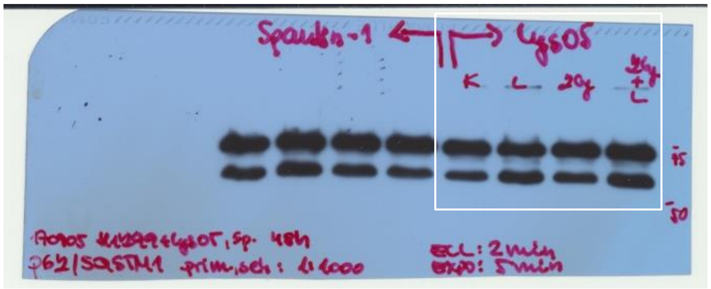

10. p62/SQSTM1; 48 h

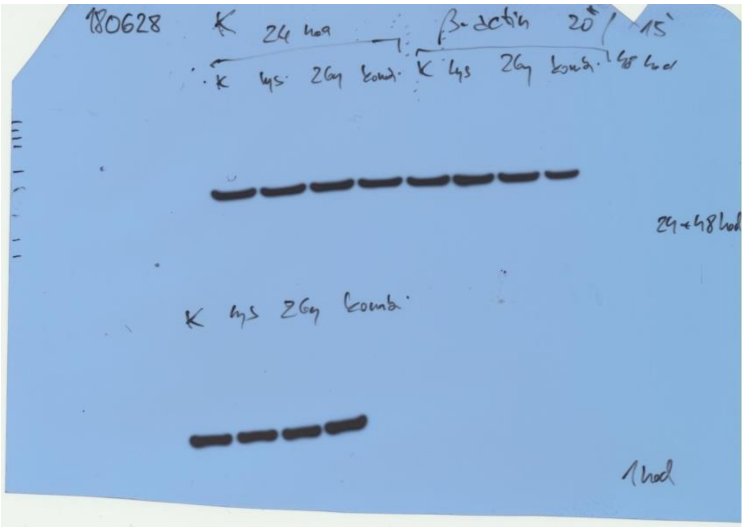

11. β-actin
